# Evaluation of an oral health scale of infectious potential using a telematic survey of visual diagnosis

**DOI:** 10.4317/medoral.18766

**Published:** 2013-03-25

**Authors:** Marta Relvas, Jacobo Limeres, Inmaculada Tomás, Cristina Cabral, Corsina Velazco, Pedro Diz

**Affiliations:** 1 School of Dentistry, Instituto Superior de Ciências da Saüde-Norte, Oporto, Portugal; 2 Grupo de Investigación en Odontología Médico-Quirúrgica (OMEQUI), Santiago de Compostela University, Spain; 3 School of Medicine and Dentistry, Santiago de Compostela University, Spain

## Abstract

Objective: To compare the results of a subjective estimation of oral health through review of a set of intraoral photographs with those of an objective oral health scale of infectious potential. Method: The pool of patients was made up of 100 adults. Using an infectious-potential scale based on dental and periodontal variables, we assigned 1 of the 4 grades of the scale (range, 0 to 3; 0 corresponds to an excellent oral health status and 3 to the poorest oral health status) to each subject. A total of 20 representative subjects were selected from the pool of patients, 5 subjects for each one of the grades of the scale, and a standardized photographic record was made. One thousand dentists practicing in Spain were sent the survey by e-mail and 174 completed forms were received. We then calculated the concordance of the oral health status indicated by the respondents after visualising the photographs on comparison with the results of the oral health scale of infectious potential; concordance was termed correct grade allocation (CGA). Results: The majority of respondents (69.1%) achieved a CGA in 8 to 12 cases and none achieved more than 15 CGAs. The poorest CGA rates were found with grades 1 and 2, with a mean of 1.74 ± 1.09 and 1.87 ± 1.18, respectively, out of a maximum of 5. The concordance in terms of CGA was high for grade 0 (70.5%), very low for grade 1 (10.8%), low for grade 2 (37.3%), and moderate for grade 3 (42.6%). Conclusion: In comparison with visual examination of the oral cavity, the use of objective scale that establishes a reliable diagnosis of oral health in terms of infectious potential was found to be advantageous.

** Key words:**Diagnosis, intraoral photographies, oral health scale, objective estimation, visual examination.

## Introduction

In recent years, numerous authors have suggested that there is a relationship between oral infections, in particular periodontal disease, and an increased risk of developing certain systemic diseases (1), most importantly those affecting the cardiovascular system (2,3), respiratory system (4) and premature deliveries (5). More diseases have recently been added to this list, including diabetes, rheumatoid arthritis, osteoporosis, cancer of the pancreas, metabolic syndrome, kidney failure, and even degenerative disorders such as Alzheimer disease (6).

This adds a new perspective to epidemiological studies, particularly in terms of causality. These epidemiological studies are not always reproducible and it is therefore difficult to extrapolate their results to other populations (7). The objective evaluation of variables such as plaque accumulation, the presence of caries, gingivitis, and periodontal disease enables the reproducibility of oral epidemiological studies to be improved and their bias minimized. This requires the dentist to perform a highly detailed intraoral examination. The gold standard for this is visual inspection and tactile examination of all the oral structures, including hard and soft tissues, using specific light sources for oral cavity examination and devices such as probes, periodontal probes and dental mirrors, and applying measures to prevent cross-infection (gloves and methodical hand washing) (8).

However, at the beginning of the 1990s, nursing textbooks started to make explicit reference to the importance of evaluation of the oral cavity. This led to the development of a simplified oral examination technique that only included procedures for the control of cross-infection and the use of an extraoral light source; this technique was elaborated mainly for nurses, doctors, auxiliary staff, and other health professionals (8). The technique, characterized by its simplicity as it was based on visual examination, was used to elaborate tools such as the Brief Oral Health Status Examination (BOHSE), which have been employed to quantify the oral health status of residents of geriatric institutions, with the added advantage that the examination could be performed by the auxiliary staff and required only minimal training (9). In subsequent empirical research on the use of this type of visual examination by trained auxiliary staff, it was demonstrated that these persons were able to identify oral health problems effectively, initiate appropriate interventions (such as referral of the patient to a dentist), and prevent or minimize the morbidity inherent to a poor oral health status (10).

In 2007 we proposed a “global oral health scale”, which we applied to determine whether the oral health status could affect the development of bacteraemia caused by dental manipulation (11). That version of the scale had certain limitations and it has therefore been progressively modified in order to increase its reproducibility, until we obtained the version of the “global oral health scale” of possible infectious potential now proposed.

The objective of the present study was to compare the results of a subjective assessment of the oral health status achieved through review of a set of intraoral photographs with a new global oral health scale that establishes a single value for the infectious potential of the oral cavity.

## Material and Methods

-Pool of patients and application of the oral health scale of infectious potential

The pool of patients was formed of 100 adults aged between 25 and 65 years, with different levels of gingival inflammation and periodontal disease. Patients were examined in the Clinical Periodontics Unit of the Northern Higher Institute of Health Sciences (Gandra, Porto, Portugal).

A single examiner reviewed the oral cavity (excluding the third molars) of all patients, inspecting 6 sites per tooth: mesiobuccal, mediobuccal, distobuccal, distolingual, mediolingual, and mesiolingual. The following variables were recorded in each patient: number of tooth surfaces with supragingival plaque (12); number of teeth with caries (diagnosed using a probe and mirror) and severity of the caries (1 = involving enamel, 2 = involving enamel and dentin, 3 = involving enamel, dentin and pulp); number of tooth surfaces with gingival inflammation (13); and mean periodontal probing depth and number of pockets ≥4 mm, determined using a manual periodontal probe calibrated at 3, 6, 8 and 11 mm (PCP 11, Hu-Frieday, Chicago, IL, USA) (the periodontal probing depth was defined as the distance between the free gingival margin and the base of the periodontal pocket).

Combining objective information recorded using these reproducible dental and periodontal indices, we have developed a new global oral health scale that is applicable to the adult population. Both the grades of dental and periodontal health correspond to that achieved by at least 2 of the 3 variables analysed in each of these categories. If there are differences between the grades assigned to 3 variables of a category, the parameters for “caries” and “pathological pockets ≥ 4 mm” will be imposed. If the same grade is granted to 2 variables of a category and the third variable is 2 levels higher, one grade higher than the matching variables is assigned. The grade of global oral health is determined by the category (dental or periodontal), which has the highest grade. We assigned 1 of the 4 grades of the scale (range, 0 to 3; 0 corresponds to an excellent oral health status and 3 to the poorest oral health status in terms of infectious potential) to each subject ( Table 1).

**Table 1:**
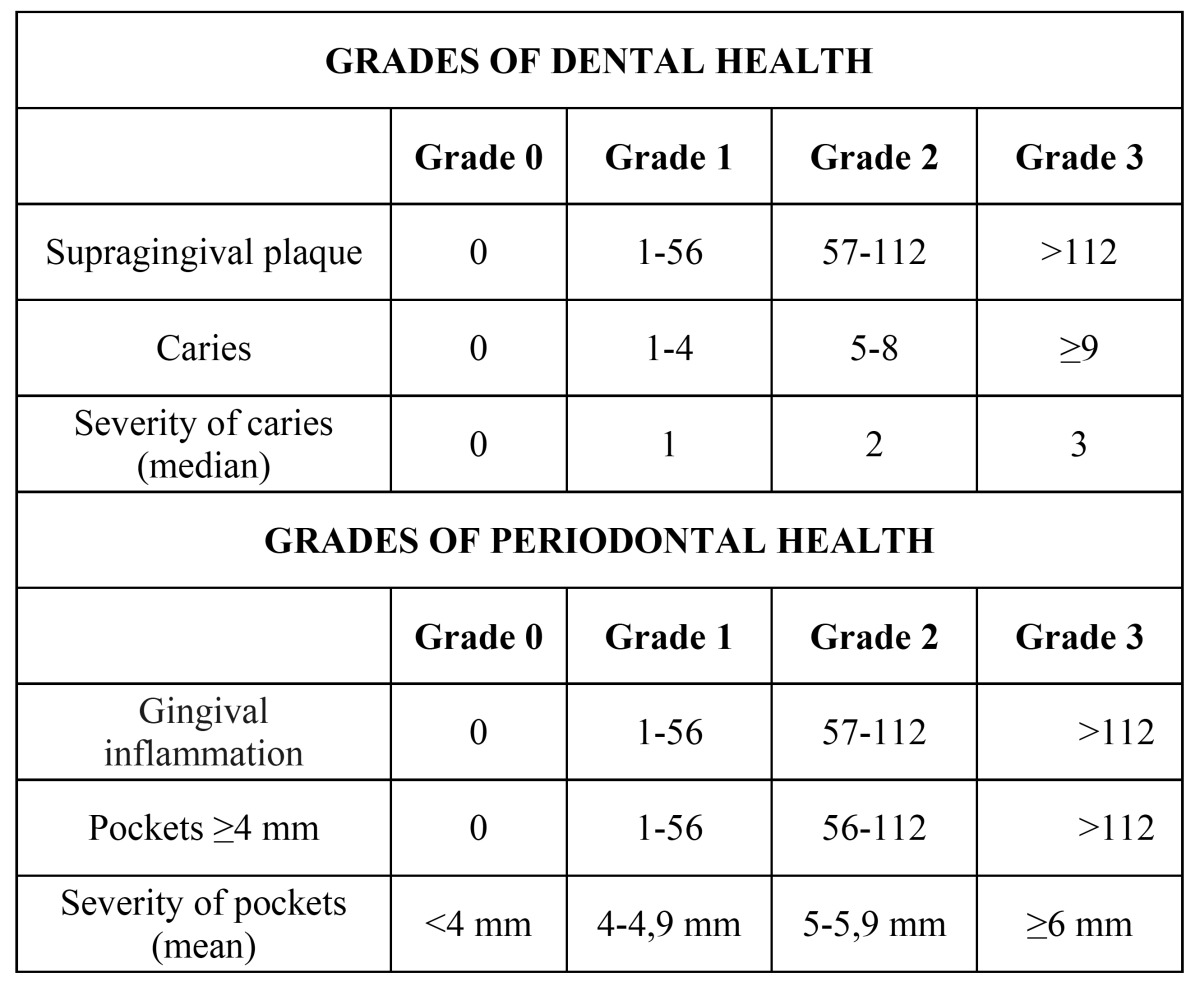
The new global oral health scale.

The project was approved by the Ethics Committee of the Instituto Superior de Ciências da Saúde Norte (Gandra, Porto, Portugal). Informed consent was obtained from all subjects before their participation in the study.

-Selection of the cases included in the survey

Twenty representative cases were selected from the pool, 5 for each one of the grades of the scale (from 0 to 3) and a standardized photographic record was produced with frontal, left lateral and right lateral with the arches in occlusion, and superior and inferior occlusal views (1 photograph of each view). The photographs were processed and filed by patient in a PowerPoint file (version 2007, Microsoft Corporation, Redmond, Washington, USA). A montage was then created with the photographs of each patient visible on a single screen. In order to perform a telematic survey, the 20 screens (corresponding to the 20 cases selected) were compressed into a single file managed using the LimeSurvey programme (The LimeSurvey Project Team, Carsten Schmitz, Hamburg, Germany).

-Distribution of the survey

Using the Puntex guide (Spanish Dentists Annuary, 2010, 37th edition, Barcelona, Spain), 1000 dentists practicing in Spain were selected at random, and were sent the survey by e-mail. The survey consisted of 2 sections: a) a number of initial questions on the socio-occupational situation of the dentist and b) evaluation of the 20 cases selected. The initial questionnaire gathered the following information on the dentist: sex, qualification (dentist or stomatologist), area of specialization (general dentistry, periodontics, orthodontics, oral surgery, cosmetic dentistry, or other), institution (private clinic; health center/hospital; university; private clinic plus health center/hospital or university; health center/hospital plus university; and other combinations); and population of the town where the respondent worked (<5,000, 5,000-20,000, 20,000-100,000, or >100.000).

The time estimated to complete the survey was 5-10 minutes. Anonymity of the respondents was guaranteed by automatic allocation of a personal record number and all respondents gave their informed consent before accessing the survey.

-Statistical analysis

Data were processed using the PASW Statistics package® (SPSS 18®) for Windows. We calculated the percentage of cases with overestimation, underestimation, and concordance of the oral health status indicated by the respondents after visualizing the photographs on comparison with the oral health status calculated using the oral health scale of infectious potential. Concordance was termed “correct grade allocation” (CGA). The mean number of CGAs in each grade was then calculated for the variables: sex, age, specialty, population of the town where the respondent worked, and institution, and the results were compared using the Kruskal-Wallis and the Mann-Whitney U tests, after confirming that the data did not have a normal distribution using the Kolmogorov-Smirnov test. A P value less than .05 was considered statistically significant. For multiple comparisons (for the variables specialties, population of the town in which the respondent worked, and institution) we applied the Bonferroni correction in order to control the type I error rate, obtaining significance levels of .003, .008, and .005, respectively. That is, 2 values of the variables specialties, population of the town in which the respondent worked, and institution differed significantly when the critical levels obtained were <.003, <.008, and <.005, respectively.

## Results

The survey was open for 6 months, during which period 174 completed forms were received (17.4% of the requests for participation issued). Just over half the respondents were women (54.7%). The mean age of the respondents was 35.2 ± 11.2 years, with a range of 22 to 66 years. The most frequent qualification was dentistry (83.9%). The majority (62.4%) worked in general dentistry, 10% in orthodontics, and 9.4% in periodontics. The majority of the respondents practiced in private clinics, either exclusively (44.8%) or else part-time (34.3%), in which case they also worked in a health center/hospital or in a university. With regard to the place of work, 44.2% of respondents worked in towns with a population >100,000 and 27.9% in towns with a population of 20,000-100,000.

After examining the photographs of the 20 cases included in the survey, the majority of the respondents (69.1%) made a CGA in 8 to 12 patients and none achieved CGAs in more than 15 cases.

For grade 0, the mean number of CGAs was 3.52 ± 1.42, and more than half of the respondents (59.1%) achieved 4 or 5 CGAs. The poorest CGA values were observed in grades 1 and 2, with means of 1.74 ± 1.09 and 1.87 ± 1.18, respectively; none of the respondents achieved a CGA in all 5 cases of grade 1, and only 1 respondent achieved this in the grade 2 cases. The mean number of CGAs in grade 3 was 2.13 ± 1.05.

Individual analysis of the cases included in the survey revealed significant discrepancies, such as overestimation by 94.2% of the respondents in case C11 (a patient with grade 1) or underestimation by 97.7% of respondents in case C14 (a patient with grade 3). To minimize this bias, the 5 cases of each grade were analyzed as a group. CGA concordance was high for grade 0 (70.5%), very low for grade 1 (10.8%), mainly due to overestimation, low for grade 2 (37.3%), also predominantly due to overestimation, and moderate for grade 3 (42.6%) ( Table 2).

**Table 2:**
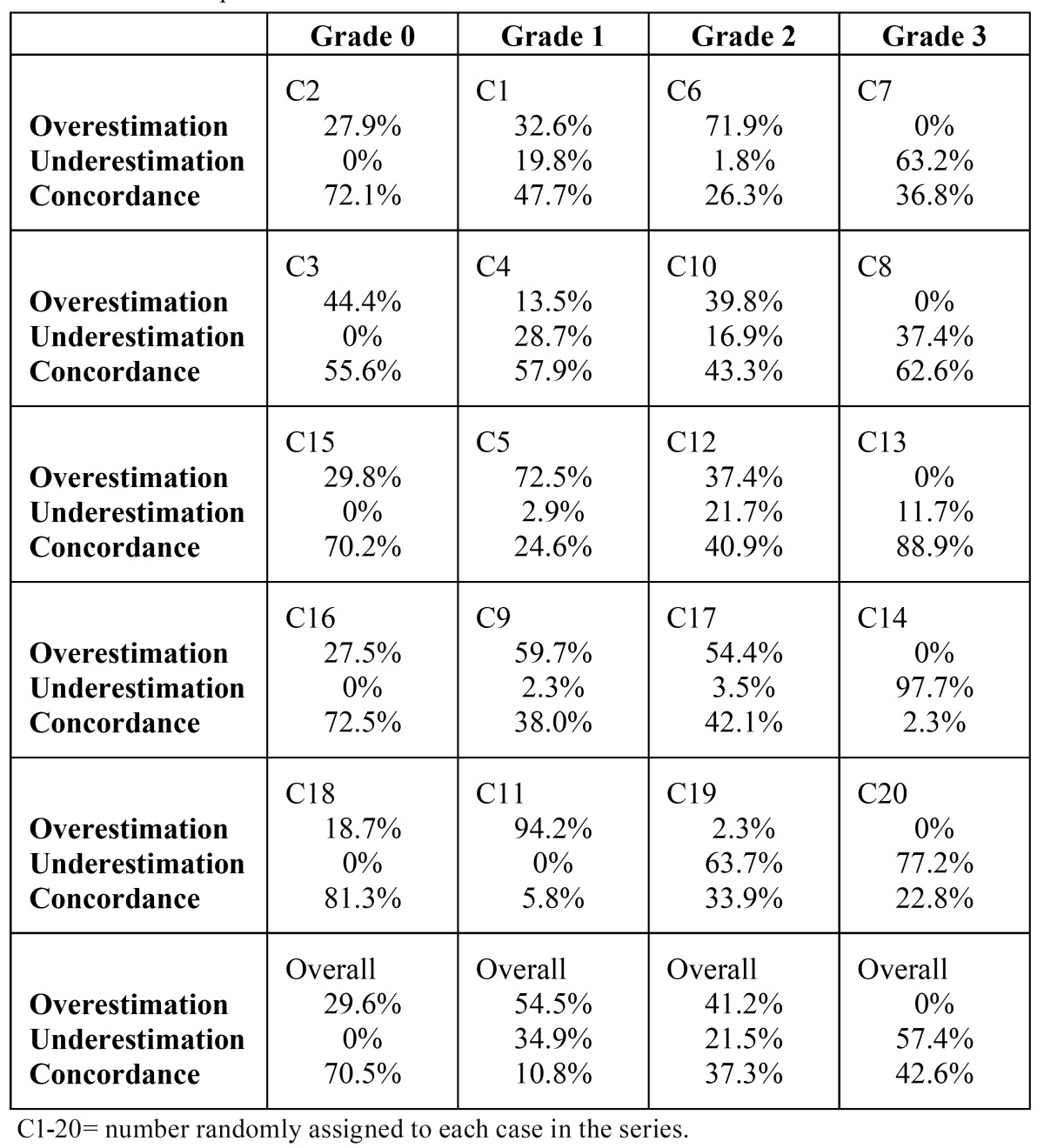
Overestimation, underestimation, and concordance between the oral health status indicated by the respondents after examining the photographs and the grade of the oral health scale of infectious potential.

The CGA was not affected by the age or qualification of the respondents or the institution or the population of the town where the respondent worked ( Table 3). Significant differences were detected with respect to the sex of the respondents: women achieved higher CGA values for grade 3 (P=.001) and men for grades 1 and 2 (P=.007 and P=.022, respectively). Statistically significant differences were also detected according to the area of specialization of the respondents ( Table 3); specifically, the mean number of CGAs for subjects with grade 1 was higher among general dentists than among orthodontists (P=.038). Differences were also observed on analysis of the mean number of CGAs for grade 2 achieved by orthodontists compared with other specialists (P=.040).

**Table 3:**
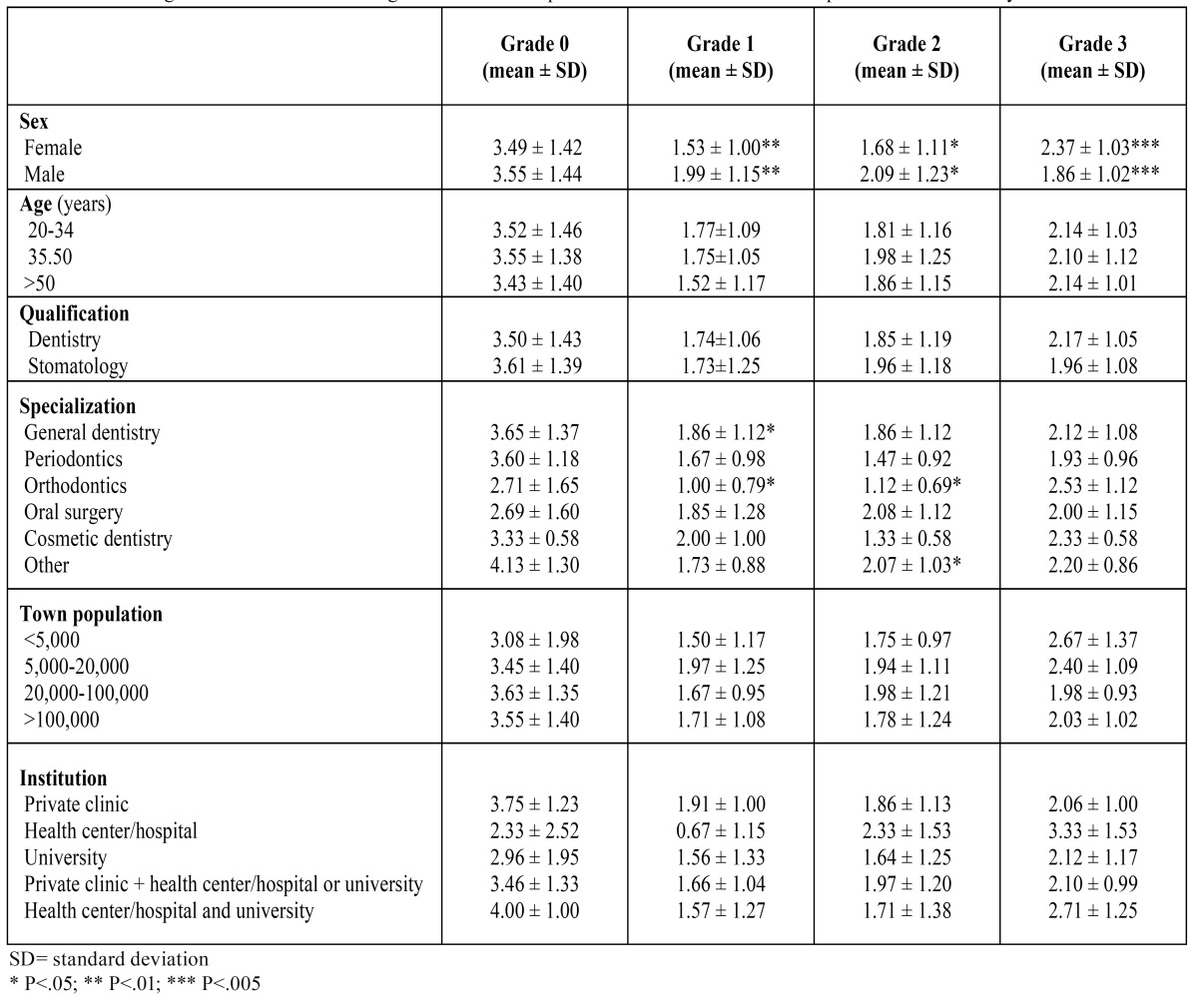
Correct grade allocation” according to the socio-occupational characteristics of the respondents in the survey.

## Discussion

In a systematic review published in 2005, Chalmers and Pearson (14) concluded that the Brief Oral Health Status Examination, based exclusively on visual examination, was the safest and most reliable validated instrument for nurses and carers to evaluate the oral health status of institutionalized persons. Other, useful but less rigorously validated instruments are also available, such as the Index of Activities of Daily Oral Hygiene and the Mucosal Plaque Score. Visual inspection has also been used successfully to determine the oral health status in children, showing good reproducibility, with sensitivity, specificity, and predictive values above 90% for the evaluation of the prevalence of caries when compared with visual-tactile (15). Although the benefits and drawbacks of applying exclusively visual criteria rather than other more complex and costly diagnostic techniques have still not been fully evaluated, the use of visual examination has become widespread and is frequently employed to monitor the clinical course of oral disease and to provide information to those responsible for health policy (16).

In the present study, clinical photographs were used as the instrument for an exclusively visual examination. This introduces a relevant bias, as the image is 2-dimensional, which can interfere with the interpretation of certain findings, and an image does not permit the use of instruments to optimize inspection of the oral cavity, such as cheek retractors and tongue depressors. Despite these limitations, series of clinical photographs of selected patients is a method frequently employed in surveys in the field of dentistry (17-19). The most common are those that directly evaluate orofacial cosmetic appearance or its affective and psychological implications (18), although they have also been used to compare treatment plans (17) and to analyze the results of orthodontic treatment (19). A library of clinical photographs enables multicenter studies of oral health to be designed, evaluating intercultural differences and including respondents with different levels of training in dentistry (20). In addition, digital images can be computer-manipulated to provide models with specific characteristics according to their facial proportions, cephalometric measurements, or occlusal patterns (21).

Apart from the methodological issues, there are certain limitations that must be taken into account when evaluating the results obtained in the present series. The first comes from the low response rate to the invitations to participate, as there may be a bias of personal or professional affinity between the respondents and the authors of the study; however, the predominance of dentists in general dental practice among the respondents is a true reflection of the current professional spectrum in Spain. The years of professional experience of the respondents was not specifically evaluated, although it may be assumed that their qualification (dentist/stomatologist) is a good indicator of this variable, as the Royal Decree establishing the qualification of Dental Specialist in Spain was passed in 1986, implying that the first dentists obtained their qualification in the early 1990s, after the elimination of stomatology as a university qualification. Nor were respondents asked about the socio-economic setting of their professional practice, and the population of the town in which the respondents worked probably does not accurately reflect this variable; however, there are no indicators that the socio-economic setting could affect the results of the survey.

Taking into account that less than 1% of the respondents achieved an overall CGA rate equal to or greater than 75%, the initial impression is that this is a poor result. However, we have not been able to find any study in the literature in which the infectious potential is evaluated on the basis of clinical photographs, and we therefore have nothing with which to compare our results. The highest CGA values corresponded to grades 0 and 3, indicating that it was possible to identify patients with an excellent or very poor oral health status in a photographic image. Poorer CGA concordances, on the other hand, were found among patients with grades 1 and 2, with a tendency to overestimation. An explanation of this result is based on the parameters that make up the oral health scale we used as reference, which included the presence of supragingival plaque, caries, gingivitis, and pathological periodontal pockets.

The clinical quantification of the accumulation of dental plaque is performed using tested indices that frequently involve the application of a dye to highlight the plaque (12). This is an unsophisticated method, as it does not enable the topography, composition, or viability of the bacteria in the plaque to be determined; more sophisticated analysis techniques have therefore been developed, such as fluorescent solutions and confocal microscopy. In the scale used as reference in the present study, the number of tooth surfaces with supragingival plaque was determined visually, without the use of highlighting agents, and the quantification of plaque accumulation in the photographic images ought therefore to be similar, or in any case it would be underestimated in both assessment methods. Consequently, plaque accumulation would not appear to be implicated in the discordance detected between the 2 assessment methods.

It has been stated that visual-tactile examination is more effective for establishing a diagnosis of caries than exclusively visual examination, though both methods underestimate non-cavitated lesions (22). Some authors have suggested that radiology increases diagnostic sensitivity for the detection of occlusal caries (23), although others state that x-rays are ineffective until the lesion is well established and affects the dentine (24). The most critical authors even maintain that the standard methods of detection of caries combining visual and tactile examination plus x-rays are limited, as they do not detect incipient bacterial activity at critical sites such as within fissures or below fillings (25). As a result, more sophisticated caries detection and quantification techniques have been marketed, such as those based on fluorescence emission or variations in electrical impedance (24). In the reference scale used in the present study, the diagnosis of caries was established using visual-tactile examination, which could lead to differences in terms of the prevalence and severity of caries with respect to lesions detected on clinical photographs. In the literature there is controversy regarding the use of probes for the diagnosis of caries (25), and it was stated in a consensus document published in 2001 that probes provide little diagnostic information and may even be counterproductive (The National Institutes of Health, Bethesda, USA. http://consensus.nih.gov/2001/2001DentalCaries115Program.pdf). After reviewing one of the cases included in the survey, case C14, we observed that the patient had many superficial caries and that the grade of infectious potential was underestimated by 97% of the respondents. This allows us to suggest that the sensitivity of purely visual examination for the diagnosis of caries is lower than that of visual-tactile examination, and this difference could be even greater if we take into account that the photographs were taken without previous toothbrushing (22).

It has been demonstrated that self-perception of periodontal disease correlates with the presence of pathological pockets (26), but there is no concordance between the symptoms reported by patients and the evaluation of photographs by health professionals (27). It is currently considered that periodontal probing depth and attachment loss constitute the best indicators for the epidemiological study of periodontal disease (28). In the reference scale used in the present study, first generation periodontal probes of the type typically employed in epidemiological studies (22) were used to determine only the mean depth of periodontal pockets and the number of pathological pockets; these variables are not sufficient to establish a diagnosis of periodontitis, but do provide information on the actual periodontal status (7). It has been suggested that the periodontal probe is an essential complement to visual inspection, both for clinical assessment of the state of the periodontal tissues and to perform epidemiological screening studies (29). This could explain the diagnostic underestimation in some cases in the present survey, such as patient C7, who had numerous, very deep periodontal pockets, and whose grade of infectious potential was underestimated by 63% of the respondents.

The only parameters that affected the CGA were sex and the area of specialization of the professionals surveyed, particularly orthodontics, although these 2 parameters probably overlap. Among general dentists, the proportion of men and women was similar and therefore this condition would not have affected the results. However, 11 (84.6%) of the 13 oral surgeons among the respondents were men and 15 (88.2%) of the 17 orthodontists were women. This tendency of male dentists to favor surgical activities and of women to favor orthodontics and pediatric dentistry has already been reported in the literature (30). It could be speculated that the CGA rates achieved by men in the cases that were more difficult to interpret (grades 1 and 2) could be due to the higher proportion of oral surgeons, who are more accustomed to diagnosing and treating infectious disease than specialists in orthodontics.

## Conclusion

In comparison with visual examination of the oral cavity, the use of objective scale that establishes a reliable diagnosis of oral health in terms of infectious potential was found to be advantageous.
